# Dr. Anandibai Joshi: An Indian Medical Pioneer

**DOI:** 10.7759/cureus.69227

**Published:** 2024-09-11

**Authors:** Reena Doomra

**Affiliations:** 1 Pharmacology, Manav Rachna Dental College, Manav Rachna International Institute of Research and Studies, Faridabad, IND

**Keywords:** anandi joshi, female physicians, gender equality, historical vignette, medical education, pioneering women, women's health

## Abstract

Dr. Anandibai Joshi was the first Indian female physician to complete her studies in Western medicine at the Women's Medical College of Pennsylvania. A pioneer in medicine, she was a trailblazing Indian woman, who had faced many hurdles during her quest for education. In her short span of life, she faced a lot of emotional, mental, physical, and social turbulence and had to prove herself at every step to the orthodox patriarchal Indian society the need to study medicine. Studying medicine in India was impossible for females during the 19th century. During those days, the females in India were uneducated due to gender differences prevalent in society, so the only option was to pursue medicine in a foreign land. Her life was full of hardships and challenges; she got married at the age of nine and, at the young age of 14, delivered a baby boy who could not survive beyond 10 days due to lack of medical care. This was the turning point in her life, and she was determined to become a physician so that the other women would not suffer the way she had. Thus began her educational journey, to pursue a career in Western medicine. Before Anandibai went to the United States, she gave a motivating speech in a public gathering, expressing the need for female doctors in India and her keenness to pursue medicine in the United States. She challenged all the social norms in those days, whether it was fighting to break gender stereotypes or convincing the male-dominated society to study medicine in the United States. This article is a reflection of Anandibai's accomplishments in her life and medical career and her dedication, determination, and strong commitment to improving the lives of Indian women. Her short life is a reflection of hope, perseverance, determination, and success and an inspiration to many Indian women to pursue education and medicine.

## Introduction and background

**Figure 1:**
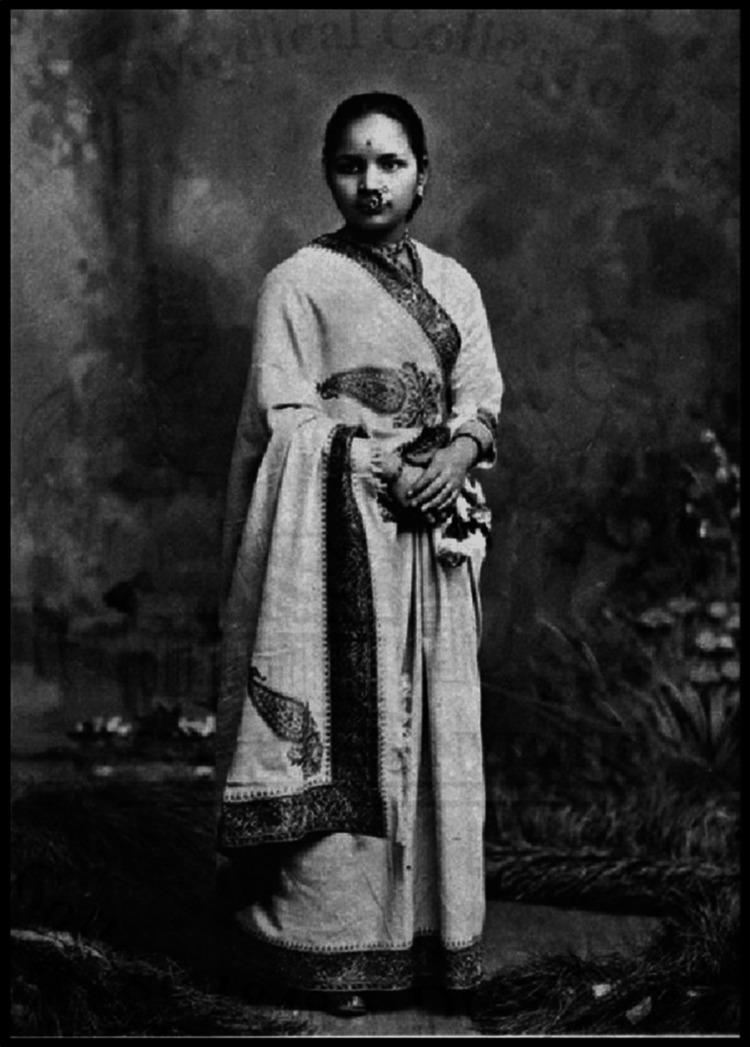
Dr. Anandibai Joshi Reference: [1]

During the 19th century, Indian society prevented women from mainstream education due to patriarchal Indian culture. Females were deprived of education due to gender bias and cultural and social barriers. Modern history has witnessed many undaunting women, women who have broken the conventional expectations from the family and society, and one among them was Dr. Anandibai Joshi who got her two-year medical degree from the United States with her sheer persistence, determined to improve the healthcare status of Indian women (Figure 1). Her life is an inspiration and motivation for all women and has paved the way for them to pursue education not only in India but in the entire South Asian subcontinent.

Dr. Anandibai Joshi, also known as Dr. Anandi Gopal Joshi, was born as Yamuna on March 31, 1865, to Gangabai and Ganpatrao Joshi in a Chitpavan Brahmin family in the Kalyan region of Bombay Presidency, Maharashtra [2]. Her parents belonged to a family of landlords and were wealthy Brahmins. Anandibai was the sixth of the 10 children. When Anandibai was a child, her father recognized Anandibai's zeal for knowledge and encouraged her. He ensured that Anandibai was educated at home in Marathi. 

As childhood marriage was a common practice in India during the 19th century, Anandibai was married to Gopalrao Joshi when she was only nine years old, who renamed her "Anandi." At the time of their marriage, Gopalrao was a post-master in Alibag, Maharashtra. He was a widower and had liberal views on female education. He always wanted Anandibai to be educated and found a sincere student in Anandibai, who was nearly 20 years younger. With an encouraging father during her childhood and a supportive husband after marriage, she could pave the way for herself in the orthodox Indian society, dominated by males. At the young age of 14, Anandibai became a mother and gave birth to a baby boy. Unfortunately, the baby could not survive and died 10 days after birth due to lack of medical care [2]. This was the turning point in her life, as she realized that timely medical attention from a female physician could have saved her newborn child. Thus, determined to become a physician and help the innumerable women in an orthodox society in India, she had a firm resolve, an unshakable faith within herself to become a physician. With continued support and encouragement from her husband and an inner urge to improve the health of Indian women, she worked hard on her education. She moved to Calcutta with her husband where she could learn English and Sanskrit. Anandibai faced all the obstacles and criticism that prevented women from studying and defied all the rules of oppression during those days. Anandibai's strong-willed character, determination, and dedication towards improving the health of women in India took her to America, the first woman from the Bombay Presidency of India to graduate with a two-year degree in Western medicine. Before leaving India, in her pursuit of knowledge, she addressed a gathering at Serampore College hall in 1883 and expressed and explained her desire to pursue medicine in the United States. She expressed her views on the lack of female doctors and the need to strengthen the healthcare status of women in India [3-6].

## Review

Medical career and achievements

Anandibai's father recognized little Anandibai's quest for knowledge and encouraged her, wanting her to be educated, which was uncommon for females during those times. Since the females were deprived of formal education, his father had a school in a portion of his house and made sure that Anandibai was educated. She was married at the young age of nine, which was a common practice in India during those days. She became a mother at 14 and delivered a baby boy. However, the newborn lived for nearly 10 days and died due to lack of medical care, emergency management, and female physicians in India. This was the turning point in Anandibai's life, a strong desire to become a doctor and serve society. She discussed with her husband who was more than willing to ensure that Anandibai could achieve her goal. As there were no educational institutes in India to permit education in medicine for females, the only option was to study medicine abroad. 

Anandibai faced a lot of hardships and opposition from society and fought against all odds. Gopalrao stood as a pillar of strength by her side and ensured and tried his best that Anandibai could study Western medicine. Many orthodox Indians stood against her as a Hindu woman was inclined to study medicine abroad. The idea itself was against the traditional norms which an Indian housewife was challenging boldly. Strongly supporting his wife, Gopalrao wrote a letter to Royal Wilder, an American missionary, to help Anandibai Joshi study medicine in the United States. Wilder published the letter in Princeton's Missionary Review. This letter was read by Theodicia Carpenter, a resident of Roselle, New Jersey, who was thoroughly impressed by Anandibai's interest to pursue medicine and her husband's support for her to do the same. For nearly two years, Theodicia and Anandibai exchanged their views regarding religion, social, and cultural differences between the two countries. During this period, Anandibai gained confidence in self-expression in English. Theodicia was instrumental in convincing Wilder, and Anandibai was lucky to get a chance to study medicine at the Women's Medical College of Pennsylvania (WMCP), Philadelphia [7-9].

Challenges and overcoming barriers

Before leaving for the United States, Anandibai gave a public speech in Serampore College hall, explaining her reasons for studying Western medicine. She mentioned that India was in dire need of female physicians and vowed to adhere to the Hindu traditions and customs in the United States. She wanted to improve the healthcare of Indian females. Her health started deteriorating, and she had a fever, breathlessness, and headache. Despite all this, with her strong will and undaunting spirit, Anandibai set on a journey to complete a Doctor of Medicine (MD) degree in 1886 from WMCP, now called Drexel University. Anandibai's colleagues included Kei Okami from Japan and Sabat Islambouli from Syria, who were also the first female physicians in their respective homeland [3].

Dr. Anandibai's thesis titled "Obstetrics among Aryan Hindus" included references from ancient Indian texts as well as from American medical literature. She also received a letter from Queen Vitoria, the Empress of India, congratulating her in recognition of her medical degree. Dr. Anandibai Joshi returned to her home country where she was given a warm-hearted welcome and greatly appreciated for her accomplishments in 1886. She created a historic milestone at such an early age and was the first Indian woman to achieve a medical degree from the West. She had been appointed as the physician-in-charge and was given the responsibility of Edward Albert Memorial Hospital in Kolhapur. However, she succumbed to tuberculosis at an early age on February 26, 1887, before becoming 22 years old [10]. She had achieved what she had set out for herself and created history by making huge strides at such a young age. She broke all the gender stereotypes and paved the way for women to seek education, study medicine, and serve society.

## Conclusions

Despite her short life, Dr. Anandibai Joshi inspired women throughout the country and is a pioneer in Indian healthcare. She was a feminist and wanted to help the females in her homeland. She was instrumental in breaking the gender bias and proved that with female education in India, the health status of women and children would improve. Her indomitable spirit and legacy continue paving the way for innumerable women to pursue education and medicine. Dr. Anandibai Joshi's life is an inspiration to fight against all odds and to achieve success. Her determination, perseverance, firm resolve, and indomitable spirit paved the way for female physicians in India. The Institute of Research and Documentation in Social Sciences (IRDS) named the Anandibai Joshi Award for Medicine in honor of her significant contribution. The Government of Maharashtra has a fellowship program in her name to honor young females working on women's health. Her indomitable spirit continues to encourage young females and women to face any challenge and adversity in life for the betterment of society.
